# Supplementary material for: Dynamic Regulation of Endogenous Transcription Factor Hubs at Single‐Molecule Resolution
Source: Adv Sci (Weinh). 2026 Jul 8:e00048. Online ahead of print. doi: 10.1002/advs.202600048 (PMC13343610; doi:10.1002/advs.202600048)
Supplement: Supplementary file 1 — Supporting File: advs76287‐sup‐0001‐SuppMat.docx. [file ADVS-9999-e00048-s001.docx]

**Supporting Information for “Dynamic regulation of endogenous transcription factor hubs at single-molecule resolution”**

Shawn Yoshida^1,2^, Yanghao Zhong^1^, Akiko Kumagai^2^, William G. Dunphy^2^, Shasha Chong^1^*

^1^ *Division of Chemistry and Chemical Engineering, California Institute of Technology, Pasadena, CA 91125, USA*

^2^ *Division of Biology and Biological Engineering, California Institute of Technology, Pasadena, CA 91125, USA*

* Correspondence: [schong@caltech.edu](mailto:schong@caltech.edu)


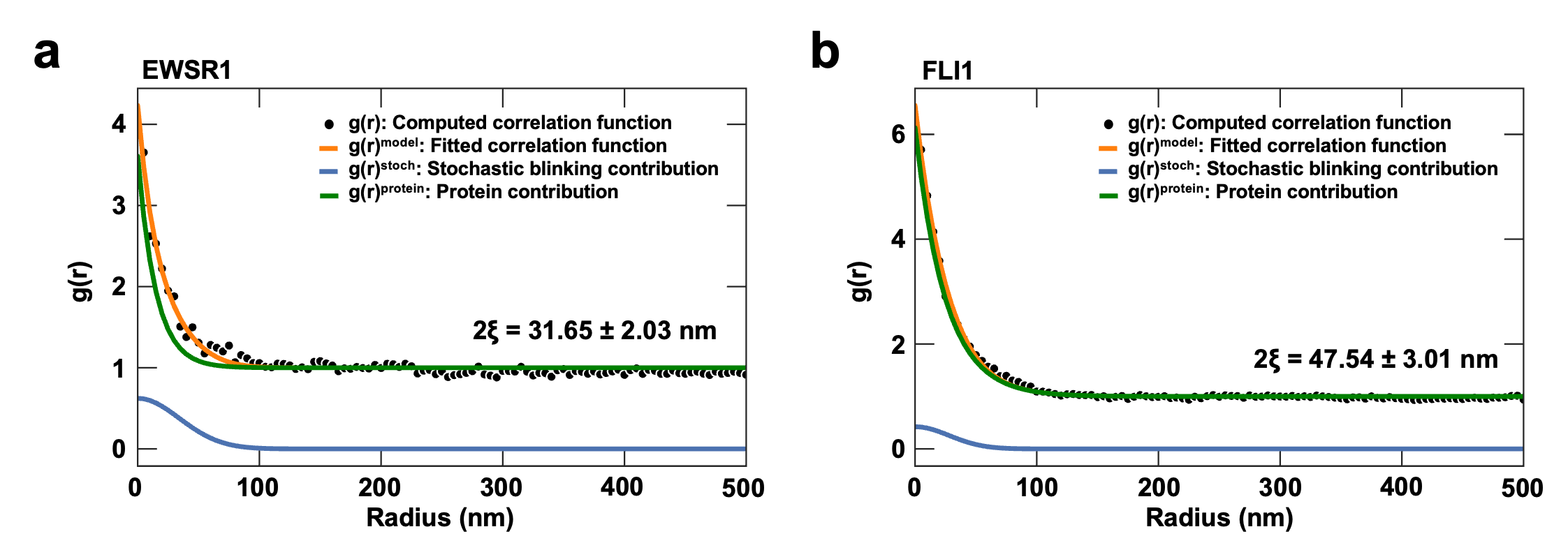


**Figure S1: Pair-correlation PALM characterizes the approximate size of EWSR1 and FLI1 puncta.** The computed pair-wise autocorrelation functions of the raw PALM localizations (black) for (a) EWSR1 and (b) FLI1 were fit to a model that accounts for the contributions of both true protein clustering and the multiple appearances of the same fluorophores due to stochastic blinking (orange). The protein correlation term and stochastic blinking correlation term are shown in green and blue, respectively. The correlation length of protein clusters, ξ, is proportional to the cluster size and 2ξ can be used as an estimate of average cluster diameter. EWSR1 and FLI1 puncta are significantly smaller than EWS::FLI1 hubs with approximate diameters 2ξ = 31.65 ± 2.03 nm and 2ξ = 47.54 ± 3.01 nm, respectively. Data is from n = 10 cells.

**
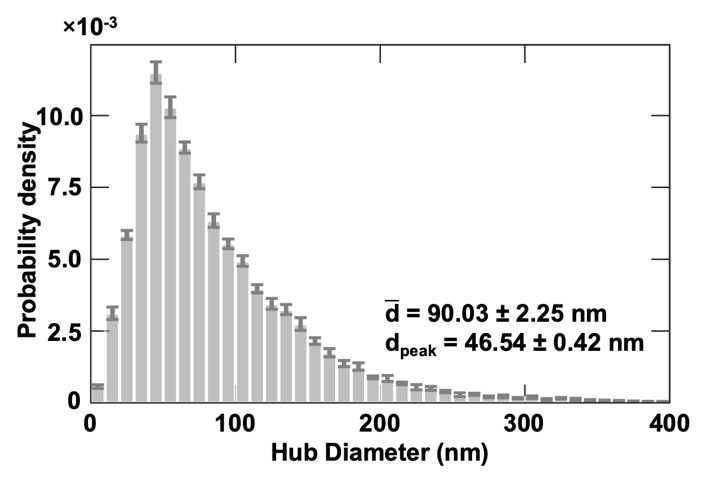
**

**Figure S2.** **PALM microscopy characterizes the dimensions of EWS::FLI1 hubs after treatment with triptolide.** The distribution of mean hub diameters generated by clustering photoblinking-corrected localizations with DBSCAN.

**Supplementary Note A:**

In the mean-field treatment of homogeneous nucleation, the free energy corresponding to the nucleation of a spherical hub of radius, $R$, is written as

| $\Delta G=4\pi R^{2}\gamma-\frac{4}{3}\pi R^{3}\nu^{-1}k_{B}T\log\left( S \right)$ | (S1) | | |
| --- | --- | --- | --- |
|  | |  |  |

where $\gamma$ is the surface energy at the interface, $\nu$ is the volume occupied by a single molecule, and $S=c/{c^{*}}$ is the supersaturation, or the ratio of the actual concentration to the equilibrium or saturation concentration. Then, for $n$ molecules, the total volume occupied by the hub is $n\nu=\frac{4}{3}\pi R^{3}$ and rearranging to obtain expression for $R$ and substituting it into Equation S1 yields

| $\Delta G={(36\pi)}^{1/3}\nu^{2/3}\gamma n^{2/3}-nk_{B}T\log\left( S \right)$ | (S2) | | |
| --- | --- | --- | --- |
|  | |  |  |

Defining $\varepsilon:={(36\pi)}^{1/3}\nu^{2/3}\gamma$ and recognizing that $\Delta\mu=k_{B}T\log\left( S \right)$, we obtain Equation 1, the compact form of Equation S1 used in the main text, Equation 1.

| $\Delta G=\varepsilon n^{2/3}-\Delta\mu n$ | (S3) | | |
| --- | --- | --- | --- |
|  | |  |  |

The critical hub size $n_{c}$, the corresponding critical radius $R_{c}$, and free energy barrier to nucleation ${\Delta G}_{c}$, are set by the maximum of the free energy profile; thus, we set $\frac{d}{dn}\Delta G=0$ and find $n_{c}=\left( \frac{2\varepsilon}{3\Delta\mu} \right)^{3}$, corresponding to

| $R_{c}=\frac{2\varepsilon}{3\Delta\mu}$ | (S4) | | |
| --- | --- | --- | --- |
|  | |  |  |

and

| ${\Delta G}_{c}=\frac{4\varepsilon^{3}}{27{\Delta\mu}^{2}}$ | (S5) |
| --- | --- |

**Supplementary Note B:**

Assuming Becker and Döring kinetics where individual molecules (rather than multimers) join and leave hubs, the steady-state nucleation rate per unit volume follows the Zeldovich form given by^1^

| $J=\rho\beta Zexp\left( \frac{-{\Delta G}_{c}}{k_{B}T} \right)$ | (S6) |
| --- | --- |

Where $\rho$ is the number density of molecules in $m^{-3}$, $\beta$ is the impingement rate of molecules in $s^{-1}$, and $Z$ is the Zeldovich factor, which accounts for the curvature of the free energy profile around the critical radius.

We can estimate that $\rho=3.20\times{10}^{19} m^{-3}$ using 200 nM the concentration of EWS::FLI1 from Chong et al. 2018^2^ with Avogadro’s number. The impingement rate for diffusion-limited (rather than reaction-limited) incorporation can be estimated as $\beta=4\pi R_{c}D\rho=77.98 s^{-1}$, where $D=1.97\times{10}^{-12} m^{2}s^{-1}$ is the diffusion coefficient of EWS::FLI1 from the main text. The Zeldovich factor is a function of the curvature of $\Delta G$ at the critical size and is given as

| $Z=\sqrt{\frac{-1}{2\pi k_{B}T}\left( \left. \frac{\partial^{2}\Delta G}{\partial n^{2}} \right\vert_{n=n_{c}} \right)}$ | (S7) |
| --- | --- |

We can rewrite this using Equation 1 yielding

| $Z=\frac{1}{3k_{B}T}\pi^{-1/2}\varepsilon^{1/2}n_{c}^{-2/3}$ | (S8) |
| --- | --- |

Notably, $Z$ (hence $J$) is not insensitive to constant multiplicative factors of $n_{c}$. Thus, rather than using the fit value, we take the average number of localizations in hubs of size $R_{c}$ and divide by $0.21$, the effective localization efficiency for PA-JF549 to estimate that $n_{c}=153.21$. Using this value of $n_{c}$ together with the fitted value $\varepsilon=0.0015k_{B}T$ yields $Z=2.54\times{10}^{-4}$. We can also use the fitted value of $\frac{{\Delta G}_{c}}{k_{B}T}=4.88$. Using our measurements with Equation S6, we estimate that $J=6.83\times{10}^{16} m^{-3}s^{-1}$.
